# Multi-Omics Analysis Reveals Concentrate Supplementation Alleviates Body Weight Loss by Regulating Rumen Function in Lactating Tibetan Sheep During the Cold Season

**DOI:** 10.3390/ani15192791

**Published:** 2025-09-25

**Authors:** Chao Yang, Qingling Ma, Jiancui Wang, Zhiyou Wang, Shengzhen Hou

**Affiliations:** 1 State Key Laboratory of Plateau Ecology and Agriculture, Qinghai University, Xining 810016, China; yangchaocas@126.com; 2 College of Agriculture and Animal Husbandry, Qinghai University, Xining 810016, China; qinglingma@126.com (Q.M.); wangjiancui2025@126.com (J.W.); wangzhiyou52@163.com (Z.W.)

**Keywords:** histomorphology, digestive enzymes, rumen fermentation, microbiota, lactating Tibetan sheep

## Abstract

The parturition season of grazing Tibetan ewes spans from October to March, a period that exacerbates the adverse impacts of nutrient-deficient herbage on milk yield, body condition, and postpartum recovery. To alleviate the weight loss of ewes during the cold seasons, we provided concentrate supplements at four levels (dry matter (DM) basis), 260 g (C1), 440 g (C2), 520 g (C3), and 610 g (C4), alongside a basal diet of grazed pasture. A total of 96 multiparous Tibetan ewes (third parity, body weight: 45.17 ± 3.69 kg (body weight (BW) were enrolled within 12–18 h postpartum and randomly allocated to four dietary groups (*n* = 24 ewes per group). We measured growth performance, ruminal histomorphology, fermentation parameters, and digestive enzymes. A multi-omics technique (16S rRNA gene sequencing and RNA-seq) was employed to investigate the mechanisms underlying alterations in ruminal function. The results showed that increasing the concentrate level decreased body weight loss and increased average dry matter intake (*p* < 0.05). Rumen morphology was significantly altered: papilla width and muscle layer thickness were greatest in the C4 group, whereas submucosal thickness was highest in the C1 group (*p* < 0.05). Cellulase activity was lowest in the C1 group (*p* < 0.05). Papilla width of lactating Tibetan ewes in the C4 group was higher (*p* < 0.05) than that in the C1 and C3 groups. Concentrate supplementation altered ruminal microbiota composition and diversity. Each group exhibited a distinct microbial signature: the C1 group was characterized by *Lachnospiraceae_XPB1014_group*, *Candidatus_Omnitrophus*, *Paenibacillus*, and *unclassified_Oligoflexaceae*; the C2 group was enriched in *Papillibacter*, *Anaerovibrio*, *V9D2013_group*, and *unclassified_Peptococcaceae*; the C3 group was characterized by *unclassified_Bacteroidales_RF16_group*; and the C4 group was characterized by *Ruminococcus*, *Pseudobutyrivibrio*, and *Mitsuokella* (*p* < 0.05). Transcriptomic analysis identified differentially expressed genes (*TRPA1*, *EPHB1*, *GATA3*, *C4*, *ABCG2*, *THBS4*, and *TNFRSF11B*) that are predominantly involved in immune regulation, signal transduction, and nutrient digestion. The results of Spearman correlation analysis showed that *Anaerovibrio* was negatively correlated with propionate (r = −0.565, *p* < 0.05). However, it was positively correlated with the ratio of acetate and propionate (r = 0.579, *p* < 0.05). Moreover, *Lachnospiraceae_XPB1014_group* was negatively correlated with cellulase (r = −0.699, *p* < 0.05) and α-amylase (r = −0.514, *p* < 0.05). These findings suggest that the increasing concentrate supplementation alleviates body weight loss in lactating Tibetan sheep by orchestrating improvements in rumen histomorphology, digestive function, altering bacteria composition, and ruminal immune and modulating host epithelial gene expression.

## Simple Summary

Systematic studies on the effects of concentrate supplementation on the rumen function and microbiology alterations in Tibetan ewes during their lactation period are limited. Our multi-omics analysis showed that higher concentrate levels significantly alleviated body weight loss by regulating rumen histomorphology, enhancing cellulase activity, and altering the abundance of key bacteria (*Lachnospiraceae_XPB1014_group*, *Anaerovibrio*, *Ruminococcus*, and *Pseudobutyrivibrio*). Furthermore, concentrate supplementation levels altered the expression of genes (*TRPA1*, *EPHB1*, *GATA3*, *C4*, *ABCG2*, *THBS4*, and *TNFRSF11B*) involved in immune regulation, signal transduction, and nutrient digestion. Correlation analysis confirmed that these changes are interconnected, as indicated by significant associations between rumen bacteria, fermentation parameters, and digestive enzyme activities. Our findings clarify that concentrate supplementation mitigates weight loss by orchestrating functional improvements across the rumen ecosystem.

## 1. Introduction

The Qinghai–Tibet Plateau—recognized as the “Roof of the World” and the “Third Pole”—is the highest and most extensive plateau on earth. Its extreme environmental conditions—including hypobaric hypoxia, severe cold, high UV-B radiation, and a low partial pressure of oxygen—impose stringent selective pressures on resident organisms [1]. Tibetan sheep (*Ovis aries*), an endemic livestock species on the plateau, are phenotypically and ecotypically stratified into three distinct topographical ecotypes: highland-, valley-, and oula-types [2]. These animals represent a critical indigenous genetic resource, having endured millennia of exposure to high-altitude, cryogenic, hypoxic, and high-irradiance conditions. Through long-term natural and artificial selection, they have evolved a suite of integrated physiological, morphological, and molecular adaptations. These include augmented erythropoiesis, blunted hypoxic pulmonary vasoconstrictive response, dense double fleece, and enhanced DNA-repair capacity, which collectively confer exceptional fitness within this harsh cryosphere environment [3].

The abbreviated pasture growing season typically leads to an insufficient nutrient supply for grazing ruminants, particularly acute from November to May, a period that consistently induces live-weight loss in Tibetan sheep within extensive grazing systems [4]. Crucially, the parturition season for grazing Tibetan ewes occurs from October to March of the subsequent year. This time exacerbates the adverse impacts of herbage nutrient deficits on both lactation performance and postpartum recovery [5]. Consequently, mitigating nutrient deficiency during lactation is a pivotal constraint on the production efficiency of the Tibetan sheep industry. Although herders routinely provide concentrate diets to lactating ewes to maximize nutrient intake, formulating the most cost-effective supplementation regimen remains a significant challenge. Moreover, the digestive efficiency of nutrients from pasture and concentrate supplements in the rumen critically impacts postpartum recovery in Tibetan ewes.

As the hallmark digestive organ of ruminants, the rumen underpins their adaptation to high-altitude ecosystems. Ruminal feed fermentation and hydrolysis are governed almost exclusively by a complex and dynamic microbial consortium [6]. This pregastric microbial ecosystem comprises bacteria, methanogenic archaea, ciliate protozoa, and anaerobic fungi, collectively mediating the degradation of dietary substrates [7]. Functionally, rumen microbes can be categorized by their primary metabolic roles, such as amylolytic, fibrolytic, and proteolytic [8]. Their catabolism yields volatile fatty acids (VFAs) and ammonia-N—key metabolites absorbed and utilized by the host. The composition and metabolic activity of this microbiome are modulated by diet composition, feeding regimen, ambient environment, health status, and host genotype [9]. Inter-species and inter-breed divergence in microbial profiles may therefore arise during environmental adaptation. For example, elevated ambient temperature increases the abundance of *Streptococcus bovis* and other amylolytic bacterial populations in Holstein cattle [10], while the same stressor reduces amylolytic populations in small ruminants [11]. In plateau-adapted herbivores, the gut—especially the rumen—harbors a highly diverse, cellulolytic microbiota that facilitates the utilization of low-quality forage and sustains short-chain fatty acid (SCFA)-dependent energy homeostasis under chronic nutrient scarcity [12]. However, the responsiveness of the rumen microbiome to graded levels of concentrate supplementation during the cold season, and the concomitant shifts in ruminal nutrient absorption and post-ruminal metabolism, remain to be elucidated.

In the current study, we used RNA-sequencing and 16S rDNA amplicon sequencing to investigate the ruminal function and the associated shifts in bacterial community structure and function in response to different levels of concentrate supplementation in lactating Tibetan sheep. Specifically, our objectives were 3-fold: (1) to quantify the extent to which incremental concentrate allowances mitigate body weight loss in lactating ewes; (2) to evaluate treatment-induced alterations in rumen histomorphometry, fermentation end-products, and the activity of key digestive enzymes; and (3) to elucidate the regulatory mechanisms by which concentrate level reshapes rumen microbial community structure and associated metabolic pathways. Our study identified the optimal concentrate inclusion rate for minimizing body weight loss and delineated the corresponding adaptive responses of rumen function and microbiota. This work aims to provide an evidence-based nutritional strategy to enhance the productivity of lactating Tibetan ewes during the cold seasons.

## 2. Materials and Methods

### 2.1. Animal and Experimental Design

The experiment was conducted at Jinzang Tibetan Sheep Breeding Professional Cooperative from October 2023 to January 2024, and all protocols were approved by the Animal Ethics Committee of Qinghai University (permit No. QUA-2023-0112).

A total of 96 multiparous Tibetan ewes (third parity, body weight (BW): 45.17 ± 3.69 kg were enrolled within 12–18 h postpartum and randomly allocated to four dietary groups (*n* = 24 ewes per group). Each group comprised three pens (replicates) with eight ewes per pen. To simulate voluntary dry matter intake (DMI) under natural grazing conditions, the metabolizable energy (ME) requirement was calculated using metabolic BW (BW0.75) according to the method described by Liu H. [13]. Grazing Tibetan sheep have been reported to consume 62.8 g dry matter (DM) kg/BW0.75 daily. Based on the mean metabolic BW of 17.37 kg recorded in the present study, the baseline DMI was estimated to be 1.10 kg DM/d per ewe, corresponding to 73.4 g crude protein (CP), 15 g ether extract (EE), and 8.18 MJ metabolic energy daily from native pasture. Accounting for the elevated nutrient demands of lactation, roughage allowance (DM basis) was increased to 1.40 kg per ewe per day (50:50 oat hay: wheat straw), isonitrogenous and isoenergetic with the above-mentioned pasture. On this basal roughage, ewes received daily concentrate supplements (DM basis) of 260 g (C1), 440 g (C2), 520 g (C3), and 610 g (C4) per ewe, respectively; ingredient composition and nutritional levels are presented in Table 1, and the ingredient composition and chemical composition of diets in each group was presented in Table S1.

Prior to the trial, all pens were thoroughly cleaned, disinfected, and equipped with creep barriers to separate lambs during concentrate feeding, enabling accurate individual recording of feed intake of the ewes. Ewes were offered feed in two equal meals at 08:30 and 16:40 h and had ad libitum access to fresh water. Lambs were temporarily removed from dams before the reception of roughage and concentrate; they were returned to the ewes only after the ewes completed consumption of the respective diets. Throughout the experimental period, lambs received exclusively the dam’s milk and were not provided creep feed or milk replacer.

### 2.2. Sample Collection

At the end of animal feeding experiment, six experimental Tibetan ewes were selected from each group (two ewes from each pen within one group) for slaughter after a fast of 12 h. Anesthetization of the selected ewes was precisely carried out via an intravenous injection of sodium pentobarbital administered at a dosage of 50 mg/kg BW. Following the induction of anesthesia, exsanguination was performed until the ewes attained a surgical plane of anesthesia. Subsequently, the abdominal cavity was carefully opened, and the rumen was dissected and separated from the reticulum. The ruminal digesta was then filtered through four layers of gauze, and the resultant rumen fluid was collected in 5 mL cryogenic vials (Corning^®^, Corning, NY, USA). The collected rumen fluid was immediately subjected to freezing using liquid nitrogen and subsequently maintained at −80 °C for subsequent determination of volatile fatty acids and digestive enzymes. Ruminal tissue of the abdominal sac was dissected and washed in sterile culture dishes (Corning^®^, NY, USA) with ice-cold DNase/RNase-free ddH_2_O (R1600, Solarbio LIFE SCIENCES, Beijing, China) until clean (3–4 times). The tissues were cut into small pieces, then a part of the tissues was transferred into 10% neutral buffered formalin for morphology observation; the other tissues were placed in a sterile sample bag (Whirl-Pak^®^, Pleasant Prairie, WI, USA), which was immediately snap-frozen in liquid nitrogen and stored at −80 °C for RNA extraction.

### 2.3. Histomorphology Analysis

The ruminal tissue samples fixed in the buffered formalin were washed and trimmed into small square pieces (0.5 mm × 0.5 mm), dehydrated in gradient ethanol, and finally embedded in paraffin wax. Five sections for each sample were sliced, installed on glass slides, and stained with eosin and hematoxylin. Three random straight papillae of each slide were selected to measure the papilla height, width, and surface area using a fluorescence microscope (Olympus, Tokyo, Japan) with a calibrated 10-fold eyepiece graticule. Furthermore, the thickness of the submucosal layer and muscle layer were measured.

### 2.4. Determination of Rumen Rermentation

The pH of ruminal fluid was assayed using a portable pH meter (PHS-1701, Shanghai Boqu Instrument Co., Ltd., Shanghai, China). Prior to measurement, meticulous calibration of the pH meter was carried out utilizing a standard buffer solution to ensure the accuracy and reliability of the pH readings. For each ruminal fluid sample, three repeated measurements were performed to obtain robust data. Regarding the determination of volatile fatty acids (VFAs) in ruminal fluid, a gas chromatography system (Agilent 7890A, Agilent Inc., Palo Alto, CA, USA) equipped with a capillary column (AE-FFAP, specifications: 30 m × 0.25 mm × 0.33 μm; ATECH Technologies Co., Lanzhou, China) was employed, following the procedures detailed in a previous study [14]. Specifically, ruminal fluid samples were thawed and subjected to centrifugation at 16,000× *g* for 10 m at 4 °C. Subsequently, 2 mL of the supernatant was carefully collected and mixed with 400 μL of 25% metaphosphoric acid. The mixture was left to stand for 4 h at 4 °C to allow for adequate precipitation of proteins and other macromolecules. After this incubation period, the sample underwent a second round of centrifugation at 16,000× *g* for 10 min at 4 °C. To an aliquot of 200 μL of the resulting supernatant, 200 μL of crotonic acid (10 g/L) was added as an internal standard. The sample was then filtered through a 0.45-μm filter to eliminate any residual particulates that might interfere with the chromatographic analysis. In terms of the gas chromatography operating conditions, the injector and detector temperatures were precisely set at 200 °C and 250 °C, respectively. The column temperature was programmed to increase from 45 °C to 150 °C at a heating rate of 20 °C/min and was maintained at 150 °C for an additional 5 min to ensure complete elution of all VFA components.

### 2.5. Measurement of Digestive Enzymes

Ruminal fluid samples were thawed at 4 °C in a refrigerator. Subsequently, the samples were centrifuged at 10,000× *g* for 5 min at 4 °C to obtain the supernatant. The activities of several key digestive enzymes in the supernatant, including cellulase, hemicellulase, α-amylase, and β-amylase, were assayed using corresponding biochemical reagent kits (AIDISHENG, Jiangsu Aidisheng Biological Technology Co., Ltd., Yancheng, Jiangsu, China). All operations were conducted in strict accordance with the manufacturer’s instructions. Finally, the optical density values were measured with a microplate reader to determine the enzyme activities.

### 2.6. 16s rDNA Sequencing and Data Analysis

Total DNA was extracted from rumen fluid samples of 24 Tibetan ewes using the *SteadyPure* Stool DNA Extraction Kit (AG21036, Accurate Biology, Changsha, Hunan, China), following the manufacturer’s protocol. The DNA concentration was determined with a Nanodrop-2000 spectrophotometer (Thermo Fisher Scientific, Waltham, MA, USA), and the quality was evaluated by 2% agarose gel electrophoresis. The V3-V4 regions of the bacterial 16S rRNA gene were amplified via PCR using the forward primer 338F (5′-ACTCCTACGGGAGGCAGCA-3′) and reverse primer 806R (5′-GGACTACHVGGGTWTCTAAT-3′) [15]. The sequencing library was constructed using the Illumina Next^®^Ultra™ DNA Library Prep Kit (New England Biolabs, Ipswich, MA, USA). Both Qubit@ 2.0 Fluorometer and Agilent Bioanalyzer 2100 systems were employed to assess the library quality. Finally, the qualified amplicon libraries were sequenced on the Illumina NovaSeq PE250 platform (Illumina, San Diego, CA, USA) to generate 150 bp paired-end reads.

The processing of raw data commenced with quality filtering using Trimmomatic (v0.33) [16]. Following this, primer sequences were identified and excised via Cutadapt (v1.9.1). Subsequently, paired-end (PE) reads were assembled using USEARCH (v10), after which UCHIME (v8.1) was employed to eliminate chimeric sequences, resulting in high-quality clean reads [17]. These clean reads were then subjected to analysis through the Divisive amplicon denoising algorithm 2 (DADA2) method within the QIIME2 framework to generate a feature table and produce de-noised sequences [18]. Each representative feature sequence was annotated against the SILVA database (v138), and feature abundance normalization was performed based on the relative abundance of each sample across various taxonomic ranks [19]. QIIME2 was utilized to compute alpha diversity indices, including ACE, Chao1, Shannon, and Simpson, as well as beta diversity values [20]. Beta diversity was quantified using the unweighted or weighted Unifrac dissimilarity metric, Bray–Curtis, and Binary Jaccard distance, and Adonis analysis was conducted to evaluate the similarity of bacterial communities among the 24 samples. Finally, the linear discriminant analysis effect size (LEfSe) tool was applied to identify the effect size of species contributing to differences between samples using the Kruskal–Wallis test with a significance level of *p* < 0.05 and a linear discriminant analysis (LDA) score threshold of >3.5.

### 2.7. RNA Isolation, Transcriptomic Sequencing, and Bioinformatics Analysis

Total RNA was extracted from approximately 100 mg of frozen tissue ground in liquid nitrogen using the SteadyPure Universal RNA Extraction Kit (AG21017, Accurate Biology, Hunan, China), following the manufacturer’s procedures. The integrity of the extracted RNA was assessed using the Agilent 2100 Bioanalyzer system (Agilent Technologies, Santa Clara, CA, USA), with an RNA Integrity Number (RIN) greater than 7.0 indicating sufficient quality for library construction. Subsequently, 1 μg of RNA per sample was utilized for cDNA library construction using the NEBNext UltraTM RNA Library Prep Kit (Illumina, San Diego, CA, USA) in accordance with the manufacturer’s recommendations. The libraries were then amplified via PCR and validated using the Agilent 2100 Bioanalyzer system (Agilent Technologies, CA, USA). Finally, the cDNA libraries were sequenced on the Illumina HiSeq2000 platform (Illumina, CA, USA) to generate high-quality, 150 bp paired-end reads.

Raw data were subjected to quality control by FAST-QC (version 0.18.0) to obtain clean data after removing adapters and low-quality reads [21]. Clean data were then mapped to the ribosome RNA (rRNA) database to remove rRNA using Bowtie2 (version 2.2.8) [22]. We used HISAT2 (version 2.1.0) to align the clean reads to the sheep reference genome (ARS-UI_Ramb_v3.0) with default parameters [23]. The mapped reads of each sample were assembled using StringTie (version 1.3.1), and the expression of genes was estimated by transcripts per kilobase of exon model per million mapped reads (TPM) [24]. Principal Component Analysis (PCA) was conducted based on expressed gene data using R (R version 3.4.2) to assess the difference among the four groups. Differentially expressed genes (DEGs) were identified by DESeq2 package using the following parameters: fold change > 2 and false discovery rate (FDR) < 0.05 [25]. The Kyoto Encyclopedia of Genes and Genomes (KEGG) pathway enrichment for DEGs was performed on KOBAS software (version 3.0) [26], and pathways on level 3 underwent variance analysis in which adjusted *p* < 0.05 was considered as a significant difference. A Short Time-series Expression Miner (STEM) analysis was conducted to capture coordinated transcriptional responses across concentrate levels, and the analytical parameters were referred to the previous study [27].

### 2.8. Statistical Analysis

Raw data of rumen histomorphology, fermentation parameters, and the levels of digestive enzymes were preprocessed using Excel 2022 software, and statistical analyses were performed by the univariate analysis of variance (ANOVA), followed by Tukey’s HSD test for multiple comparison in IBM SPSS Statistics 25.0. All data are shown as the mean ± standard error of the mean (SEM), and *p* < 0.05 was considered to be statistically significant.

## 3. Results

### 3.1. Growth Performance of Lactating Tibetan Sheep

According to the data in Table 2, the initial BW differed significantly (*p* < 0.05) among the experimental groups, and the BW on D34 and D68 did not affect by concentrate supplementation. The extent of average BW loss during the study period was decreased (*p* < 0.05) with increasing concentrate supplementation levels. Average dry matter intake was increased (*p* < 0.05) with increasing concentrate supplementation levels.

### 3.2. Histomorphological Analysis of Rumen

**Figure 1:**
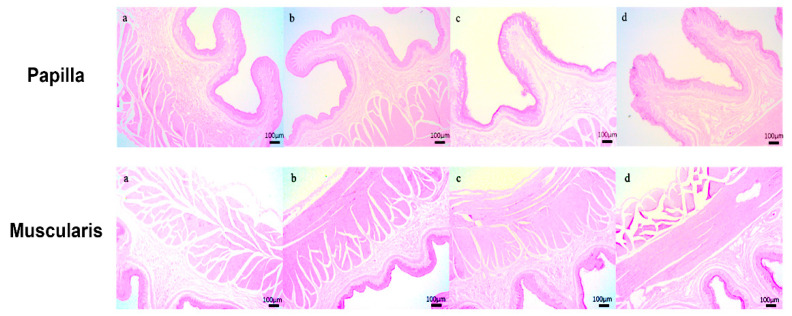
Rumen histomorphology of lactating Tibetan ewes (4×). (**a**–**d**) Rumen of lactating Tibetan ewes in the C1, C2, C3, and C4 group, respectively.

As shown in Table 3 and Figure 1, the level of concentrate supplementation significantly affected (*p* < 0.05) the rumen histomorphology, papilla width, submucosal thickness, and muscle layer thickness in lactating Tibetan ewes. Specifically, the papilla width was higher (*p* < 0.05) in the C4 group than that in the C1 and C2 groups. Conversely, submucosal thickness was higher (*p* < 0.05) in the C1 group than other groups. For muscle layer thickness, the C4 group exhibited a greater value (*p* < 0.05) than both the C1 and C2 groups, while the value in the C3 group was also higher (*p* < 0.05) than that in the C1 group.

### 3.3. Rumen Fermentation Parameters

The level of concentrate supplementation had no significant effect (*p* > 0.05) on ruminal pH, molar ratio of acetate, propionate, butyrate, isobutyrate, valerate, isovalerate, total VFA, or the ratio of acetate and propionate (Table 4).

### 3.4. Ruminal Digestive Enzyme of Lactating Tibetan Sheep

The activity of ruminal digestive enzymes, including hemicellulose, α-amylase, and β-amylase, was not significantly affected (*p* > 0.05) by the levels of concentrate supplementation (Table 5). However, the activity of cellulase in the C1 group was lower than that in the C2 group (*p* < 0.05).

### 3.5. Microbial Composition of Rumen in Lactating Tibetan Sheep

**Figure 2:**
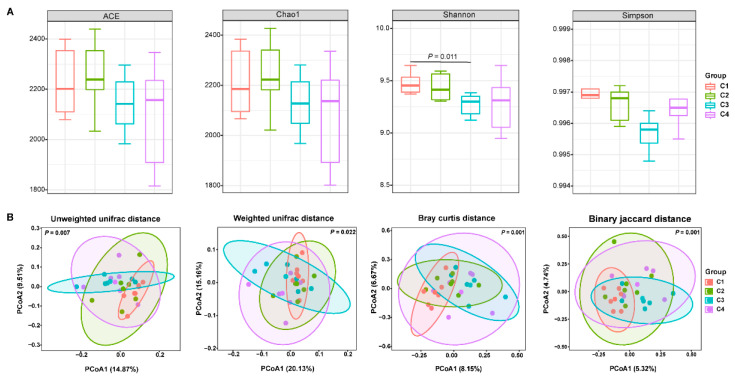
Rumen bacteria diversity of lactating Tibetan ewes. (**A**) Alpha diversity indices, including ACE, Chao1, Shannon, and Simpson. (**B**) Principal coordinates analysis (PCoA) based on unweighted or weighted Unifrac distance metric, Bray–Curtis, and Binary Jaccard distance metric, and *p* < 0.05 represents the significant difference of analysis of similarities (ANOISM).

16S rRNA sequencing of ruminal fluid samples from the four ewe groups produced 1,876,149 raw reads. Following quality control, 1,723,117 clean reads were obtained, with an average of 71,797 clean reads per sample. A total of 36,068 amplicon sequence variants (ASVs) were generated following quality control. The rarefaction curves confirmed that the sequencing data coverage was adequate to describe the ruminal microbial composition of lactating Tibetan ewes (Figure S1). To evaluate the effects of concentrate supplementary levels on ruminal microbiota, we assessed α-diversity and β-diversity. The α-diversity analysis revealed that concentrate supplementation levels did not affect ACE, Chao1, and Simpson indices. However, the Shannon index was higher (*p* < 0.05) in the C1 group than that in the C3 group (Figure 2A). Principal coordinate analysis (PCoA) based on unweighted and weighted UniFrac, Bray–Curtis, and Binary Jaccard distances revealed distinct clustering among the four treatment groups. This separation was confirmed to be statistically significant by ANOSIM analysis (*p* = 0.007, *p* = 0.022, *p* = 0.001, and *p* = 0.001, respectively; Figure 2B).

**Figure 3:**
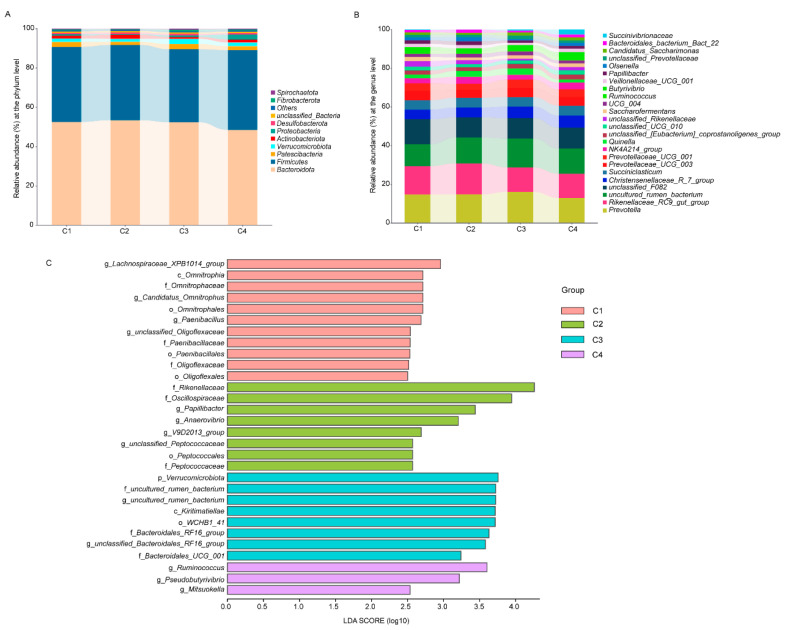
Rumen bacteria composition of lactating Tibetan ewes supplemented different concentrate levels. (**A**) Bacteria composition and the relative abundance at the phylum level. (**B**) Bacteria composition and the relative abundance at the genus level. (**C**) The representative bacteria in each group analyzed by the linear discriminant analysis effect size (LEfSe) analysis (*p* < 0.05 and linear discriminant analysis (LDA) > 3.5).

Taxonomic analysis identified a total of 22 phyla across the treatment. Six of these had an average abundance greater than 1%, with *Bacteroidota*, *Firmicutes*, and *Patescibacteria* being the most dominant phyla (Figure 3A). At the genus level, 24 genera exhibited an average relative abundance above 1%. The dominant genera included *Prevotella*, *Rikenellaceae_RC9_gut_group*, *unclassified_F082*, *Christensenellaceae_R_7_group*, *Succiniclasticum*, *Prevotellaceae_UCG_003*, and *Prevotellaceae_UCG_001* (Figure 3B). LEfSe analysis identified specific taxa enriched in each treatment group. The C1 group was characterized by a higher relative abundance of *Lachnospiraceae_XPB1014_group*, *Candidatus_Omnitrophus*, *Paenibacillus*, and *unclassified_Oligoflexaceae*. The C2 group was enriched in *Papillibacter*, *Anaerovibrio*, *V9D2013_group*, and *unclassified_Peptococcaceae*. The C3 group showed a higher relative abundance of *unclassified_Bacteroidales_RF16_group*. The C4 group was characterized by a higher relative abundance of *Ruminococcus*, *Pseudobutyrivibrio*, and *Mitsuokella* (*p* < 0.05 and LDA > 3.5; Figure 3C).

### 3.6. Transcriptomic Profile of Rumen in Lactating Tibetan Sheep

**Figure 4:**
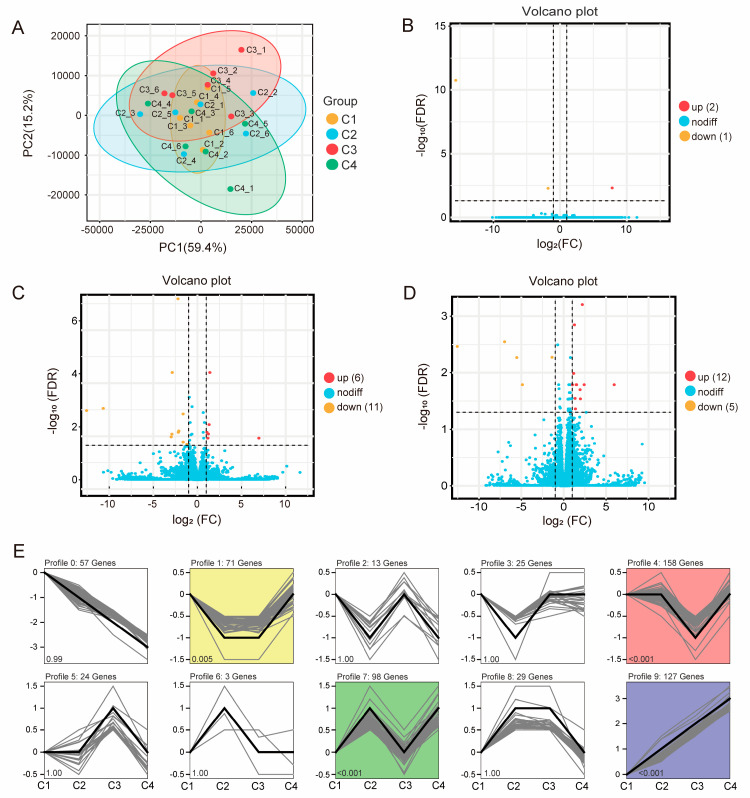
Rumen transcriptome profile of lactating Tibetan ewes supplemented different concentrate levels. (**A**) Principal component analysis (PCA) of rumen transcriptome profile. (**B**–**D**) Differential expressed gene (adjusted *p* < 0.05 and |log_2_(Fold Change)| > 1) identification between C2 vs. C1, C3 vs. C1, and C4 vs. C1. (**E**) Short Time-series Expression Miner (STEM) analysis of rumen transcriptome.

To elucidate the impact of graded levels of concentrate supplementation on the ruminal transcriptome, we performed high-throughput RNA sequencing, yielding 1.026 billion high-quality paired-end reads (4.28 million reads per sample). Of these, 89.28% were successfully mapped to the sheep reference genome. We defined expressed genes as those with transcripts per million (TPM) > 1 in at least 50% of the samples within any treatment group; this criterion retained 12,858 genes for downstream analyses. Principal component analysis (PCA) revealed no clear segregation among the four dietary groups (Figure 4A). Differential expression analysis identified 3, 17, and 17 differentially expressed genes (DEGs) in the C2 vs. C1, C3 vs. C1, and C4 vs. C1 comparisons, respectively (Figure 4B–D). The limited number of DEGs precluded robust functional enrichment of these individual gene sets.

**Figure 5:**
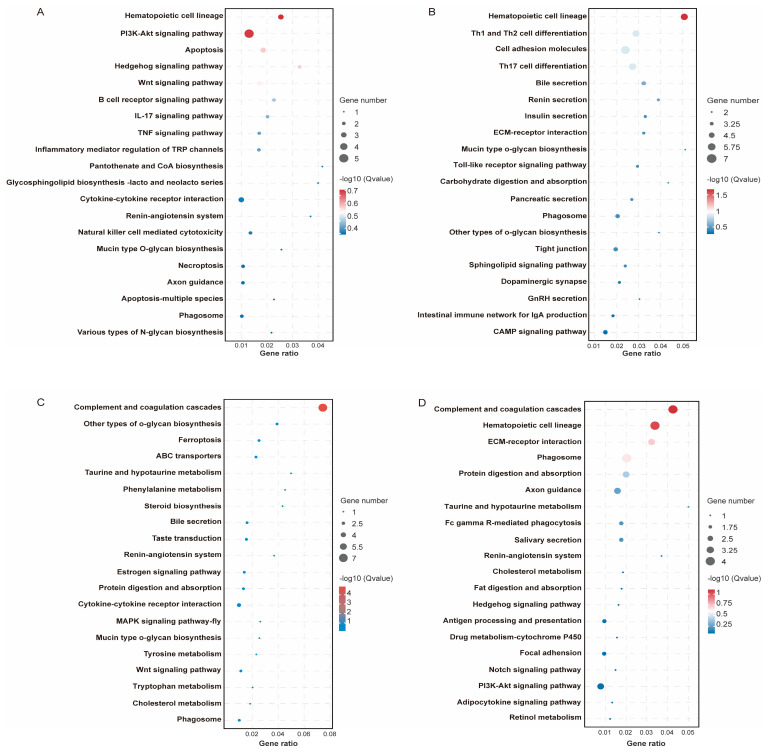
Kyoto Encyclopedia of Genes and Genomes (KEGG) pathway enrichment analysis of the genes in the profile 1 (**A**), profile 4 (**B**), profile 7 (**C**), and profile 9 (**D**).

To capture coordinated transcriptional responses across concentrate levels, we performed a STEM analysis, yielding nine distinct gene cluster profiles (Figure 4E). Among these, four exhibited statistically significant temporal patterns (*p* < 0.05): profile 1 (*n* = 71), profile 4 (*n* = 158), profile 7 (*n* = 98), and profile 9 (*n* = 127). Kyoto Encyclopedia of Genes and Genomes (KEGG) pathway enrichment showed that genes in profile 4 were significantly enriched in hematopoietic cell lineage (*p* < 0.05; Figure 5B), whereas genes in profile 7 were enriched in complement and coagulation cascades (*p* < 0.05; Figure 5C). No significant enrichments were detected for profiles 1 or 9 (*p* > 0.05; Figure 5A,D). Notably, representative DEGs—including TRPA1 and EPHB1 (profile 1), GATA3 (profile 4), C4 and ABCG2 (profile 7), and THBS4 and TNFRSF11B (profile 9)—are predominantly implicated in immune regulation, signal transduction, and nutrient digestion, suggesting that concentrate supplementation modulates ruminal function through these biological processes.

### 3.7. Correlation Analysis Between Phenotype and Bacteria

**Figure 6:**
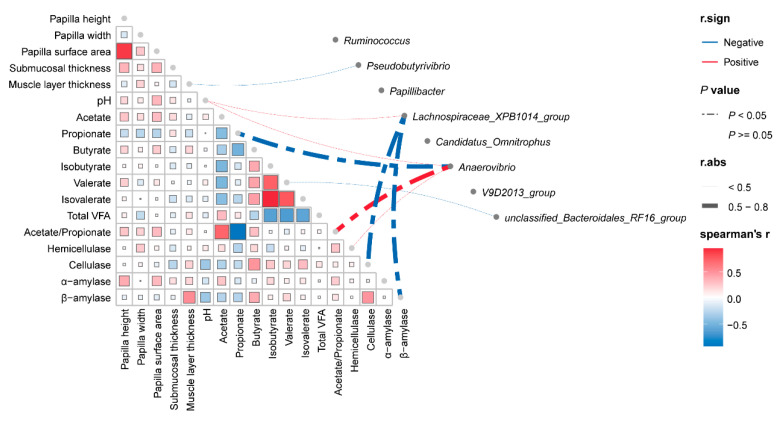
Spearman correlation analysis of the phenotype (rumen histomorphology indicators, fermentation parameters, and digestive enzyme activity) and ruminal bacteria. |r| > 0.5 and *p* < 0.05 are considered significant correlations. Read dashed line represents positive correlation and blue dashed line represents negative correlation.

We performed Spearman correlation analysis to investigate the relationship between the phenotype (rumen histomorphometric indicators, fermentation parameters, and digestive enzyme activity) and ruminal bacteria (representative bacteria in each group generated by LEfSe analysis) on OmicStudio tools https://www.omicstudio.cn/tool (accessed on 13 August 2025). The results showed that the relative abundance of *Anaerovibrio* was negatively correlated with propionate (r = −0.565, *p* < 0.05) but positively correlated with the ratio of acetate and propionate (r = 0.579, *p* < 0.05; Figure 6). Moreover, *Lachnospiraceae_XPB1014_group* was negatively correlated with cellulase (r = −0.699, *p* < 0.05) and α-amylase (r = −0.514, *p* < 0.05).

## 4. Discussion

Extreme temperature is considered one of the main environmental stresses affecting animal production, reproduction, physiology, and health [28]. On the Qinghai Tibetan Plateau, cold season temperatures range from −10 to −30 °C. These harsh conditions commonly induce a negative energy balance in Tibetan sheep, especially in lactating ewes, thereby seriously impairing lactation performance and postpartum recovery [29]. Thus, identifying effective nutritional strategies to ensure adequate energy intake for Tibetan sheep during the cold season is crucial.

Several studies have focused on dietary intervention, such as protein levels [30], concentrate-to-forage ratios [31], and dietary types [32], on the growth performance of growing Tibetan sheep during the cold season. However, few studies have explored nutritional regulation to alleviate body weight loss in lactating Tibetan ewes or their underlying regulatory mechanism. Previous research indicates that Tibetan sheep supplemented with oat hey (800 g DM/d) exhibited lower body weight loss and higher nitrogen retention and energy utilization during the winter season than those fed only native herbage [33]. Similarly, supplementing with 250 g/d of concentrate has been shown to reduce body weight loss and increase milk yield of Tibetan ewes during the cold season [34]. In this study, the increase in concentrate level significantly decreased body weight loss of lactating Tibetan ewes; the BW of those on D34 and D68 was similar. However, the BW of lambs on D60 significantly increased with increasing concentrate levels (Table S2), indicating that higher concentrate intake enhanced the availability of protein, fat, and energy, which may promote the utilization of energy and other nutrients and ameliorate negative energy balance, thereby alleviating body weight loss and supporting lactation and postpartum recovery.

The ruminal epithelium is organized into leaf-like papillae that constitute both the primary absorptive interface and a critical barrier against translocation of luminal microbes or their metabolites [35]. Quantitative morphology of papillae (height, width, and absorptive surface area) is widely accepted as a proxy for the epithelial absorptive capacity for volatile fatty acids (VFAs) and other fermentation end-products. Previous studies have demonstrated that the level of concentrate supplementation modulates papillar morphogenesis [36]; the present observation that papillar width in lactating Tibetan sheep increased with concentrate level corroborates this relationship. Histologically, the ruminal epithelium comprises, from lumen to serosa, the stratum corneum, stratum granulosum, stratum spinosum, and stratum basale. This stratified structure collectively governs VFA metabolism, vectorial transport, and barrier function [37]. The thickness of the epithelium directly influences nutrient absorption. Furthermore, the observed decrease in the thickness of the submucosal layer may be attributed to the increased production of VFAs and other metabolites, which can drive adaptive changes in morphology in rumen adaptation to improve the efficiency of the absorption of nutrients and maintain the ruminal homeostasis. A diet of coarse, withered grass typically promotes development of the rumen muscle layer, as increased ruminal motility is required to degrade and digest its high cellulose and hemicellulose content [36]. In this study, although roughage intake was similar across the treatments, the muscle layer thickness increased with increasing concentrate levels. It suggests that the greater energy availability from concentrate fermentation may promote the need for the vigorous ruminal motility required to digest forage, potentially leading to a relative enhancement in muscular development.

The rumen microbiota plays a crucial role in food digestion, growth and development, energy balance, and immune regulation in ruminant animals [38]. Ruminal fermentation parameters are key indicators underlying differences in ruminant growth performance. Among them, VFAs are fermentation products of microorganisms in the rumen, and their production depends on diet and the rumen microbiota [39]. In the previous study, the ruminal fluid samples were obtained 3–4 h after the morning feeding using a stomach tube attached to an electric pump in Tibetan sheep under barn feeding, and Tibetan sheep that received different concentrate-to-forage ratio diets had similar ruminal pH, VFA concentration, and acetate-to-propionate ratio [40], which was inconsistent with our results. In this study, the molar ratio of VFAs and pH of ruminal fluid were not affected by concentrate supplementation. This lack of significant difference may be attributed to the extended interval between feeding and sample collection (fasting 12 h before sample collection), during which VFAs were absorbed by the ruminal epithelium. In addition, the above results might be due to the ability of the rumen system that can adapt to appropriate dietary concentrate levels through the self-adjustment of rumen microorganisms [41]. *Lachnospiraceae_XPB1014_group* is an important bacterial taxon within the gut microbiota. This group can decompose pectin-like substances and degrade complex polysaccharides such as galacturonic acid, producing short-chain fatty acids (SCFAs) such as acetic acid and butyric acid that provide energy for the host [42]. In this study, *Lachnospiraceae_XPB1014_group* was negatively correlated with cellulase and α-amylase. This is consistent with its specialized role in pectin degradation, rather than in breaking down cellulose or starch. The changes in bacterial genera such as *Papillibacter* in the rumen of dairy cows are closely related to the rumen acidification environment. Under high-energy diet conditions, the rumen pH of dairy cows will decrease, which then causes a significant reduction in rumen *Papillibacter* abundance [43]. *Papillibacter* is a known butyrate producer [44], and its relative abundance is often indicative of the butyrate production levels. However, in our study, the relative abundance of *Papillibacter* was higher in the C2 group than other groups, yet the molar ratio of butyrate and pH in the C2 group did not differ from the other groups. *Anaerovibrio* is a well-known rumen lipolytic bacterium whose fermentation end-products are propionate and succinic acid, and its relative abundance was higher in the sheep fed a mixed ration than in those fed a forage-only diet [45]. The higher relative abundance of *Anaerovibrio* in the C2 group indicates that Tibetan ewes in this group may possess a greater capacity for fat degradation. Furthermore, *Anaerovibrio* was negatively correlated with propionate and positively correlated with the ratio of acetate and propionate. These results are contradictory to its function and warrant further study. The *V9D2013_group*, a unique bacterial genus in the gastrointestinal tract, degrades cellulose to produce butyrate and inhibits inflammation and oxidative stress [46]. The abundance of *V9D2013_group* was higher in the C2 group than other groups. Meanwhile, the activity of cellulose was higher in the C2 group, indicating that *V9D2013_group* may enhance cellulolytic activity to facilitate butyrate production. The relative abundance of family *Peptococcaceae* is possibly correlated with energy intake and conversion, as well as butyrate production [47]. The increased abundance of *unclassified Peptococcaceae* in the C2 group suggests that members of this taxon likely contribute to enhanced butyrate production.

In the current study, *Ruminococcus*, *Pseudobutyrivibrio*, and *Mitsuokella* were identified as key bacterial genera influenced by the dietary treatments. The genus *Ruminococcus* comprises obligate anaerobic, Gram-positive fibrolytic symbionts that ubiquitously inhabit the mammalian gut and enzymatically depolymerize diverse plant cell-wall polysaccharides [48]. Moreover, *Ruminococcus gnavus* cross-feeds on the malto-oligosaccharides and transient glucose released by *Ruminococcus bromii* during starch hydrolysis, channeling these metabolites to acetate [49]. These results indicate that *Ruminococcus* plays a comprehensive role in carbohydrate metabolism, contributing to the degradation of both structure and starch. *Pseudobutyrivibrio*, a genus within the Firmicutes phylum, effectively degrades hemicellulose and produces butyrate [50]. The major fermentation products of *Mitsuokella* are lactate, succinate, and acetate, and it is unlikely that this genus directly contributed to the butyrate production in the rumen [51]. In the C4 group, Tibetan ewes received 620 g of concentrate per day in addition to 1.4 kg of roughage. This increased concentrate likely provides more fermentable substrates, thereby directly facilitating the proliferation of *Ruminococcus*, *Pseudobutyrivibrio*, and *Mitsuokella*.

The gene expression pattern of rumen epithelium is largely affected by dietary transition. The rumen epithelial transcriptome showed a high number of genes that were differentially expressed genes in cattle exposed to rumen acidosis when feeding a high-starch diet. These genes impacted critical biological pathways, specifically those responsible for cell signaling and morphogenesis [52]. When ruminants experience malnutrition during the cold season, the expression of genes involved in carbohydrate metabolism and energy conversion was upregulated [53]. In the current study, the increased concentrate supplementation mainly affected the genes involved in immune regulation, signal transduction, and nutrient digestion. This suggests that greater nutrient intake provides abundant fermentable substrates, leading to increased production of VFAs and other end-products. These metabolites subsequently support rumen development and maintain homeostasis by modulating immune responses, signal transduction pathways, and nutrient metabolism.

## 5. Conclusions

Concentrate supplementation effectively alleviated the body weight loss in lactating Tibetan sheep experiencing the extreme temperatures of the cold season. Increased concentrate levels induced adaptive rumen morphological changes, specifically increased papilla width, and decreased submucosal and muscle layer thickness, which collectively enhance nutrient digestion and absorption. Meanwhile, concentrate supplementation altered the abundance of key rumen bacteria (*Lachnospiraceae_XPB1014_group*, *Candidatus_Omnitrophus*, *Paenibacillus*, *Papillibacter*, *Anaerovibrio*, *V9D2013_group*, *unclassified_Bacteroidales_RF16_group*, *Ruminococcus*, *Pseudobutyrivibrio*, and *Mitsuokella*), modulating the activity of digestive enzymes to optimize rumen fermentation and energy harvest for maintaining normal metabolism. These dietary changes also triggered adaptive shifts in rumen epithelium, promoting homeostasis through regulation of immune, signal transduction, and nutrient metabolism. We conclude that supplementing with 610 g (DM basis) of concentrate is an effective strategy to reduce weight loss, improving rumen morphology and improving fermentation efficiency in lactating Tibetan ewes during the cold season.

## Figures and Tables

**Table 1:** Ingredient composition and nutritional levels in concentrates for Tibetan ewes.

Item	Content
Ingredient, % DM basis	
Corn	32.00
Wheat	7.00
Palm meal	30.00
Soybean meal	4.00
Rapeseed meal	18.00
Cottonseed meal	2.00
Glucose	2.00
Medical stone	1.20
Sodium chloride	1.10
Sodium bicarbonate	0.35
CaHPO_4_	0.35
Premix ^1^	2.00
Total	100
Nutritional level ^2^	
Dry matter, %	88.00
Digestible energy, MJ/kg	10.44
Crude protein, %	14.63
Ether extract, %	2.97
Neutral detergent fiber, %	26.66
Acid detergent fiber, %	13.53
Calcium, %	0.95
Phosphorus, %	0.55

^1^ Prvided per kilogram of premix: CuSO_4_ 0.25 g, CoSO_4_ 0.08 g, MnSO_4_ 0.67 g, ZnO 0.83 g, Na_2_SeO_3_ 0.17 g, Ca(IO_3_)_2_ 0.25 g, Vitamine A 145,000 IU, Vitamine D_3_ 34,000 IU, Vitamine E 41.67 mg, antioxidant iron 0.83 g, ammonium nicotinate 83.33 mg; ^2^ Digestible energy is calculated based on the ingredients of the diet and their digestible energy content; other values of nutrients are determined by the Association of Official Analytical Chemists (AOAC) method.

**Table 2:** Growth performance of lactating Tibetan sheep.

Item	Group				SEM	*p*-Value
	C1	C2	C3	C4		
Initial BW, kg	45.75 ^a^	45.26 ^a^	44.95 ^b^	43.92 ^b^	5.542	0.013
D34 BW, kg	41.57	44.78	44.86	44.68	4.641	0.543
D68 BW, kg	37.39	44.30	44.81	45.25	4.457	0.381
Average BW loss, kg	−0.123 ^b^	−0.014 ^a^	−0.002 ^a^	0.020 ^a^	0.083	<0.001
Average dry matter intake, kg	1.19 ^d^	1.37 ^c^	1.56 ^b^	1.72 ^a^	0.164	<0.001

BW, body weight; SEM, standard error of the mean. ^a,b,c,d^ Means of a row with no common superscript are significantly different (*p* < 0.05).

**Table 3:** Rumen histological and morphological parameters of lactating Tibetan ewes.

Items	Group				SEM	*p*-Value
	C1	C2	C3	C4		
Papilla height, μm	930.01	947.49	1065.39	1054.08	461.70	0.612
Papilla width, μm	456.26 ^b^	510.35 ^b^	530.53 ^ab^	646.35 ^a^	226.09	0.008
Papilla surface area, mm^2^	423.26	520.76	574.66	569.71	353.64	0.339
Submucosal thickness, μm	423.37 ^a^	355.92 ^b^	354.44 ^b^	365.25 ^b^	113.19	0.027
Muscle layer thickness, μm	1193.13 ^c^	1219.41 ^bc^	1398.37 ^ab^	1579.55 ^a^	435.72	<0.001

^a,b,c^ Means of a row with no common superscript are significantly different (*p* < 0.05).

**Table 4:** Rumen fermentation parameters of lactating Tibetan ewes.

Item	Group				SEM	*p*-Value
	C1	C2	C3	C4		
pH	7.12	7.22	6.95	7.12	0.048	0.254
Acetate, %	68.80	68.30	66.70	65.99	0.505	0.158
Propionate, %	17.19	17.04	19.17	16.72	0.364	0.060
Butyrate, %	8.62	9.27	9.13	11.48	0.486	0.163
Isobutyrate, %	2.15	2.18	1.99	2.24	0.080	0.747
Valerate, %	0.86	0.81	0.78	0.91	0.026	0.341
Isovalerate, %	2.38	2.39	2.23	2.62	0.094	0.569
Total VFA, mmol/L	33.87	44.75	46.79	40.08	1.932	0.074
Acetate/Propionate	4.01	4.04	3.52	3.97	0.411	0.087

**Table 5:** The activity of digestive enzymes in the rumen of lactating Tibetan sheep.

Item	Group				SEM	*p*-Value
	C1	C2	C3	C4		
Cellulase, μg/h/mL	4.19 ^b^	8.66 ^a^	7.39 ^ab^	7.78 ^ab^	0.613	0.042
Hemicellulase, nmol/min/mL	60.38	78.36	55.24	59.69	3.634	0.107
α-amylase, μg/min/mL	316.26	328.17	349.96	336.29	9.278	0.655
β-amylase, mg/min/mL	2.16	2.70	4.19	5.96	0.602	0.101

^a,b^ Means of a row with no common superscript are significantly different (*p* < 0.05).

## Data Availability

The datasets presented in this study can be found in online repositories. The transcriptomics and 16s rDNA sequence data of this study have been submitted to the Sequence Read Archive (SRA) database, and the data are accessible through SRA Series accession number PRJNA1305533 and PRJNA1305115.

## Supplementary Materials

The following supporting information can be downloaded at: https://www.mdpi.com/article/10.3390/ani15192791/s1. Table S1: Ingredient composition and chemical composition of diets in each group; Table S2: Body weight changes in lambs in each group; Figure S1: The rarefaction curves and Shannon curves of each Tibetan sheep rumen fluid sample.
