# Supplementary material for: Multi-Omics Analysis Reveals Concentrate Supplementation Alleviates Body Weight Loss by Regulating Rumen Function in Lactating Tibetan Sheep During the Cold Season
Source: Animals (Basel). 2025 Sep 25;15(19):2791. doi: 10.3390/ani15192791 (PMC12523614; doi:10.3390/ani15192791)
Supplement: Supplementary file 1 [file animals-15-02791-s001.zip › animals-3852584-supplementary.pdf]

Table S1 Ingredient composition and chemical composition of diets in each group

Composition (DM basis)	C1	C2	C3	C4
Concentrate, g	260	440	620	800
Oat hay g	420	420	420	420
Wheat straw, g	980	980	980	980
Total, g	1660	1840	2020	2200
Nutrients intake				
Digestive energy, MJ/d	15.06	16.69	18.32	19.95
Crude protein, g/d	130.97	145.18	159.38	173.58
Ether extract, g/d	23.24	25.76	28.28	30.80
Neutral detergent fiber, g/d	881.13	976.67	1072.22	1167.76
Acid Detergent Fiber, g/d	654.54	725.51	796.486	867.46
Calcium, g/d	9.79	10.86	11.92	12.98
Phosphorus, g/d	5.64	6.26	6.87	7.48

Table S2 Body weight changes of lambs in each group

Items	Group				SEM	*p*-value
	C1	C2	C3	C4		
D1 BW, kg	4.87	4.36	4.14	4.3	0.124	0.133
D30 BW, kg	8.92	8.55	9.13	9.43	0.152	0.197
D60 BW, kg	11.63[c]	12.60[c]	13.66[b]	14.76[a]	0.293	<0.001

BW, body weight.

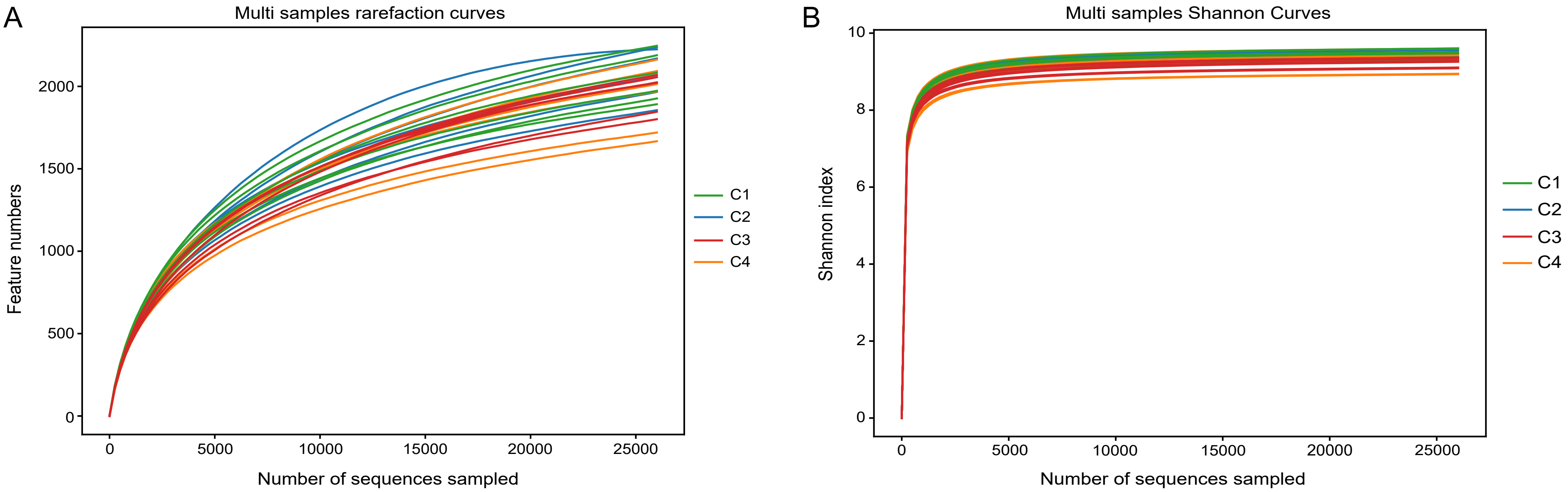

Figure S1 The rarefaction curves (A) and Shannon curves (B) of each Tibetan sheep rumen fluid sample.
